# Caveolin1 interacts with the glucocorticoid receptor in the lung but is dispensable for its anti-inflammatory actions in lung inflammation and Trichuris Muris infection

**DOI:** 10.1038/s41598-019-44963-0

**Published:** 2019-06-12

**Authors:** G. Caratti, T. Poolman, R. J. Hurst, L. Ince, A. Knight, K. Krakowiak, H. J. Durrington, J. Gibbs, K. J. Else, L. C. Matthews, D. W. Ray

**Affiliations:** 1 Faculty of Biology, Medicine, and Health, University of Manchester and Manchester Academic Health Sciences Centre, Manchester, M13 9PT UK; 2 Department of Endocrinology, Manchester Royal Infirmary, Manchester University Foundation Trust, Manchester, M139WL UK; 3 Leeds Institute of Medical Research, Faculty of Medicine and Health, University of Leeds, Leeds, LS9 7TF UK; 4 NIHR Oxford Biomedical Research Centre, John Radcliffe Hospital, Oxford, UK and Oxford Centre for Diabetes, Endocrinology and Metabolism, University of Oxford, Oxford, OX37LE UK; 5 Present Address: Institute of Comparative Molecular Endocrinology, University of Ulm, Ulm, 89081 Germany; 6 Present Address: Walter Brendel Centre of Experimental Medicine, Ludwig-Maximilians-University of Munich, Munich, 81377 Germany

**Keywords:** Acute inflammation, Protein-protein interaction networks

## Abstract

Glucocorticoids (Gcs) are widely prescribed anti-inflammatory compounds, which act through the glucocorticoid receptor (GR). Using an unbiased proteomics screen in lung tissue, we identified the membrane protein caveolin -1 (Cav1) as a direct interaction partner of the GR. In *Cav1* knockout mice GR transactivates anti-inflammatory genes, including *Dusp1*, more than in controls. We therefore determined the role of Cav1 in modulating Gc action in two models of pulmonary inflammation. We first tested innate responses in lung. Loss of *Cav1* impaired the inflammatory response to nebulized LPS, increasing cytokine/chemokine secretion from lung, but impairing neutrophil infiltration. Despite these changes to the inflammatory response, there was no *Cav1* effect on anti-inflammatory capacity of Gcs. We also tested GR/Cav1 crosstalk in a model of allergic airway inflammation. *Cav1* had a very mild effect on the inflammatory response, but no effect on the Gc response – with comparable immune cell infiltrate (macrophage, eosinophils, neutrophils), pathological score and PAS positive cells observed between both genotypes. Pursuing the Th2 adaptive immune response further we demonstrate that *Cav1* knockout mice retained their ability to expel the intestinal nematode parasite *T.muris*, which requires adaptive Th2 immune response for elimination. Therefore, Cav1 regulates innate immune responses in the lung, but does not have an effect on Th2-mediated adaptive immunity in lung or gut. Although we demonstrate that Cav1 regulates GR transactivation of anti-inflammatory genes, this does not translate to an effect on suppression of inflammation *in vivo*.

## Introduction

Glucocorticoids are widely used to treat inflammatory and immune diseases, with 1% of the UK population having a regular prescription^1^. Their action is mediated through binding and activating the glucocorticoid receptor (GR; encoded by *Nr3c1*), a member of the nuclear receptor superfamily^2^. The un-liganded GR resides predominantly in the cytoplasm, and undergoes rapid nuclear translocation in response to ligand binding^3^. The cytoplasmic location of GR is unusual, with most nuclear receptors predominantly nuclear. Some of the rapid cellular responses to Gc have been attributed this cytoplasmic location and non-genomic signalling, and cytoplasmic interacting partners have been identified such as PI3K^4^.

Once in the nucleus, the ligand-bound GR binds to cis elements in the genome to regulate gene expression^5^. The selection of gene regulation is cell type specific^6,7^, and dependent on state, for example the GR cistrome in macrophages varies in response to inflammatory activation^8^. GR can both transactivate and transrepress gene transcription, with the two modes of action being intensively studied. GR transactivation requires homodimeric GR binding to palindromic response elements (Gc response elements), but GR mediated repression is more complex. Multiple mechanisms have been reported to explain GR repression, ranging from competition of binding site occupancy, co-binding with other transcription factors, tethering to other transcription factors, binding to distinct negative Gc response elements, and induction of NFκB inhibitory genes such as *Gilz*, and *Dusp1*^9–15^. Gc treatment however, comes with some severe side-effects, including osteoporosis, type-II diabetes, and cachexia. Due to the potent anti-inflammatory capacity of GR there is interest in discerning how to harness the benefits of this therapy, but without the side-effects^16^.

Caveolin-1 (Cav1) is an integral membrane protein, involved in the formation and maintenance of caveolae. Caveolae are small membrane lipid raft domains that form flask shaped invaginations in the plasma membrane and are hubs for signalling molecules and kinases^17,18^. Cav1 is known to be involved in inflammatory signalling and can regulate the activity of NF-κB^19^. Previously we have identified an interaction between Cav1 and GR *in vitro*, and found that Cav1 regulates the non-genomic effects of Gcs^4^. Additional reports have also highlighted a role of Cav1 in regulating the genomic actions of Gcs^20^, or a direct regulation of Cav1 expression via Gcs^21^. Cav1 knockout mice have a clear lung phenotype, with hypercellularity and increased fibrosis around bronchioles^22^.

Lung inflammatory diseases, such as asthma, impose a massive human burden, and although systemic and topical Gc therapies are widely used there is variable response, and frequently high doses of potent Gc are required for effects^23^. Little is known about the principal Gc target cells in the lung, or factors within target cells which may affect the actions of applied Gc drugs. Some evidence exists to support acquired Gc resistance in response to cigarette smoking, but the precise causative chemical agent, and mechanism of action remain poorly defined. With new technology, it is now possible to look for GR-interacting proteins and to identify novel candidate GR modulators. Here we identify caveolin-1 as a ligand-dependent GR interactor, and go on to show that Cav1 regulates GR action in the lung. We find Cav1 expression to be highly variable across lung cell types, and identify differences in GR response in Cav1 null macrophages, compared to whole lung explant cultures. We go on to identify Cav1 regulation of acute pulmonary inflammation, but in contrast we found no effect on Th2 dominated allergic inflammation in the lung or Th2 mediated anti-helminth immunity in the gut.

## Materials and Methods

### Animal work

Experimentation was performed on mouse strains C57BL/6J (WT) from Harlan Blackthorn, UK and B6.Cg-*Cav1*^*tm1Mls*^/J (Cav1 KO) from Jackson Laboratories, Maine, USA. WT mice were backcrossed with Cav1 KO mice. Mice were aged between 10 and 24 weeks. All procedures were completed in compliance with Animals (Scientific Procedures) Act of 1986, and approved by the University of Manchester ethical committee. Mice had free access to food and water and were housed in a 12-hour light/dark cycle. All experiments were performed on mixed sex cohorts.

### Aerosolized LPS challenge

Age matched C57BL/6J and Cav1 KO mice were pre-treated with dexamethasone (1 mg/kg, intraperitoneal) or vehicle (cyclodextrin) for 1 h then exposed to aerosolized lipopolysaccharide (0127:B8; 1 mg/ml) or vehicle (saline) for 20 min. The animals were returned back to their cages for 5 h before sacrifice (pentobarbital, intraperitoneal). The lungs were lavaged using 1 ml BAL fluid (PBS, 10 mM EDTA and 1% BSA). Cells were quantified using Casey Counter (Schärfe System, Germany), and cytospins were stained with Leishman’s eosin-methylene blue (VWR) to enable quantification of macrophages and neutrophils.

### Ovalbumin challenge

Age matched C57BL/6J (Harlan Blackthorn, UK) and Cav1 KO mice were sensitized to ovalbumin using an adjuvant (10 μg ovalbumin, 2 mg Aluminum Hydroxide per mouse, Sigma, UK) injected intraperitoneal on days 0 and 14. Mice were then given intraperitoneal dexamethasone (Sigma, UK) or vehicle (Cyclodextrin, Sigma, UK) 3 hours before intranasal dosing of ovalbumin (1 mg/ml, 50 ul) on days 24, 25 and 26. On day 27, mice were sacrificed via pentobarbital, intraperitoneal.

### Trichuris muris infection

Age matched C57BL/6J and Cav1 KO were infected by oral gavage with a high dose (200) embryonated *T.muris* eggs. The infection was maintained by subcutaneous injections of corticosterone (50 mg/kg) or saline on days 7, 9 and 13 post infection. Adult worms were recovered from the cecum of sacrificed animals from day 20 post infection and quantified by microscope at 8x magnification.

### Analysis of GR interactome

Interactome studies were performed as done previously^24^, in brief, immunoprecipitation of endogenous GR was developed using the mouse M20 GR antibody (Santa Cruz). In order to minimize background from protein A/G used in standard immunoprecipitation procedures, antibodies were conjugated to epoxy-derivatized M270 Dynabeads (Life Technologies). 7 µg antibody/mg beads were incubated in 100 mM sodium phosphate, 1 M ammonium sulfate for 24 hours. Antibody bead complexes were then washed once in each of the following, 10 mM Tris [pH 8.0], 100 mM glycine-HCl (pH 2.5), 100 mM triethylamine, PBS and then four times in PBS with 0.1% tween.

Tissue samples were homogenized in IP lysis buffer (20 mM Bicine, 3 mM MgCl2, 250 mM NaCl, 100 mM potassium acetate, 1 µM CaCl2, 1 µM ZnCl2, 0.05% triton x100, 0.01% tween 20, phosphatase inhibitor cocktail (Phosphostop, Roche) and protease inhibitor cocktail (Promega). Samples were then incubated with 500 units of benzonase (MerckMillipore) for 15 min. Samples were spun at 16 000 × g for 15 min and antibody/bead complexes were (1 mg) added for 1 hour with constant mixing. GR immune complexes were then washed with lP-lysis buffer three rapid washes in IP buffer, followed further six times (with mixing) with IP-lysis buffer, with a new microtube used at each wash step. GR and interacting protein was eluted from the beads using 0.1% CHAPS, 0.1%SDS, 150 mM NaCl, 1 mM EDTA, 1 mM DTT and 10 mM ammonium hydroxide. Samples were electrophoresed by SDS-PAGE, stained using coomassie safestain and analyzed by Liquid Chromatography Mass Spectrometry/Mass Spectrometry.

Interactome data was processed using STRING^25^, a functional enrichment web-based platform for identifying protein-protein interactions. The interactome data is based on Gene Ontology terms, protein family database and KEGG pathway analysis.

### Liquid Chromatography-Mass Spectrometry/Mass spectrometry

Mass spectrometry was performed as in^24^. In brief, Protein bands were excised, destained with repeated incubation in 200 mM ammonium bicarbonate, 40% [v/v] acetonitrile. Gel pieces were dried with three washes in 100% acetonitrile and then trypsinized (Trypsin resuspended in 100 mM ammonium bicarbonate, 5% [v/v] acetonitrile) overnight at 37 °C. Peptides were extracted from the gel pieces by incubation in 50% [v/v] acetonitrile, 0.1% [v/v] formic acid, peptides were desiccated and resuspended in 3% [v/v] acetonitrile, 0.1% [v/v] formic acid, 20 mM citric acid; pH 2.7. 10% of the peptide sample was loaded onto a nanoACQUITY UPLC Symmetry C18 Trap (5 µm, 180 µm × 20 mm) with a flow of 15 µl/min of 3% [v/v] acetonitrile, 0.1% [v/v] formic acid and 20 mM citric acid for 5 min. Analytical separation of the peptides was performed using nanoACQUITY UPLC BEH C18 Column (1.7 µm, 75 µm × 250 mm). Briefly, peptides were separated over a 91 minutes solvent gradient from 3% [v/v] acetonitrile, 0.1% [v/v] formic acid to 40% [v/v] acetonitrile, 0.1% [v/v] formic acid on-line to a LTQ Orbitrap Velos (Thermo). Data was acquired using an information dependent acquisition (IDA) method where, for each cycle one full MS scan of m/z 300–1700 was acquired in the Orbitrap at a resolution of 60,000 at m/z 400 with an AGC target of 106. Each full scan was followed by the selection of the 20 most intense ions, CID and MS/MS analysis was performed in the LTQ. Selected ions were excluded from further analysis for 60 seconds. Ions with an unassigned charge or a charge of +1 were rejected.

Data were analyzed using Mascot (Matrix Sciences) the parameters were; Uniprot database, taxonomy *Mus Musculus*, trypsin with up to 1 missed cleavage allowed, variable modification were oxidized methionine, phosphorylated serine, threonine and tyrosine and the peptide tolerance of 0.025 Da and 0.03 Da for MS/MS tolerance.

### Flow cytometry

Flow Cytometry was performed as in^26^. Cells were isolated from lung 5 hours post LPS challenge via lavage with BAL fluid (PBS, 1 mM EDTA, 2% BSA). All cell counts were normalized to volume recovered, which ranged between 50 and 75% of initial lavage volume. Fc receptors were blocked (1:100 anti-CD16/32, eBioscience #14-0161) before application of the following antibodies in 30 μl FACS buffer (PBS, 1% BSA and 0.1% sodium azide): CD11b-PerCP-Cy5.5 (1:200), CD11c-APC (1:400,), Ly6G-FITC (1:100), Siglec-F-PE (1:400) (all purchased from eBioscience). After washing, cells were resuspended in 50 μl FACS buffer and fixed by addition of an equal volume of 3.6% formaldehyde for 20 min. Cells were resuspended in FACS buffer, and analysis was carried out on a BD LSR II flow cytometer. Neutrophils were identified as CD11b^+^Ly6G^+^. Alveolar macrophages were identified as CD11c^+^CD11b^lo^Ly6G^−^, eosinophils were identified as Siglec-F^+^CD11b^lo^.

### Immunoblot analysis

Western blot analysis was performed as previously^4^. In brief, lung tissue was collected and protein prepared in FastPrep-24 lysing matrix tubes (MP Biomedicals) then lysed using Radio-Immunoprecipitation Assay (RIPA) buffer (50 mM TricCl pH 7.4, 1% NP40, 0.25% sodium deoxycholate, 150 mM NaCl, 1 mM EDTA) containing protease (Calbiochem, San Diego, CA, USA) and phosphatase inhibitors (Sigma-Aldrich Corp.). 20 μg protein was run on an SDS 8–12% Tris-Glycine gel (Novex, Life Technologies) and then transferred onto a 0.2-μm nitrocellulose membrane (BioRad) overnight, blocked with 1% milk and probed for GR (1:1,000, clone M-20, sc-1004, Santa Cruz), Cav1 (1:1000, clone N20, Santa Cruz), Cavin (1:1000), actin (1:1000), TFIIB (1:1000, Santa Cruz) used to confirm nuclear lysis, Histone 3 (1:1000, Cell Signalling). Immunoreactivity was visualized using enhanced chemiluminescence (GE Healthcare). Densitometry was performed using ImageJ.

### Enzyme linked immunosorbent assay and mutliplex analyses

*T. muris* excretory/secretory antigen was diluted to 5 μg/ml in 0.05 M carbonate bicarbonate buffer, pH 9.6 and used to coat a 96 well plate overnight at 4 °C. The plate was washed in PBS 0.05% v/v Tween 20 (PBS-T) and non-specific binding was blocked using 3% w/v BSA in PBS by incubating at 37 °C for 1 hour and washed with PBS-T 5 more times. Supernatant were added to the plate in serial dilutions (1:20 to 1:2560) then incubated for 1 hour at 37 °C. The plate was washed 5 times in PBS-T. Biotinylated primary antibodies (rat anti-mouse IgG1 and rat anti-mouse IgG2c) were diluted in PBS-T, added to the plate and incubated for 1 hour at RT. The plate was washed 5 times in PBS-T before streptavidin-conjugated peroxidase was added then incubated for 1 hour at RT. The plate was washed 5 times in PBS-T before addition of 3,3′,5,5′-Tetramethylbenzidine (TMB). Once color had developed the stop solution (HCl) was added and the plate was read at 405 nm with a 490 nm reference.

BAL fluid was isolated and cells removed via centrifugation. BAL fluid was then stored at −80 °C before CXCL5 was quantified using CXCL5 mouse ELISA (R&D Systems) the manufacturer’s protocol. BAL fluid cytokines were also analysed by multiplex analysis using Bio-Plex Pro Mouse Cytokine 23-plex Assay (BioRad) according to manufacturer’s protocol.

### Alveolar macrophage isolation

Following cervical dislocation, lungs from WT and Cav1 KO mice were lavaged with 1 ml RPMI-1640 (Sigma). The media was then centrifuged at 1500 rpm for 5 minutes. The pellet was washed and resuspended in RPMI-1640. After 2 hours incubation, non-adherant cells were washed off with RMPI-1640. Adherent cells were cultured in RMPI-1640 supplemented with 10% FBS and 1% v/v penicillin and streptomycin (Invitrogen). The next day, cells were treated with 100 ng/ml LPS or LPS + 100 nM dexamethasone (Sigma) for 6 hours, before RNA isolation.

### RNA Extraction

Samples were lysed, and total RNA was prepared using SV Total RNA Isolation System (Promega). RNA quality was checked using the RNA 6000 Nano Assay, RNA samples with a 260:280 nm ratio of ~2 taken forward for analysis.

### Two-Step qRT-PCR

Total RNA was reverse transcribed to cDNA using High Capacity RNA to cDNA kit (Applied Biosystems) and subjected to qPCR using SYBR Green (KAPA Biosystems) detection in a q-PCR machine. All samples were analyzed in duplicate. The mRNA expression levels of GILZ, DUSP1, FKBP5 and actin were measured using appropriate primer sets (Eurofins). Expression levels were calculated using the ΔΔCT method normalizing to actin control.

### Histology and immunohistochemistry

Lungs were inflated with 4% PFA and fixed overnight before processing and paraffin embedding. 5-μm sections were cut and mounted onto slides.

Colon was cut into 10 mm sections, removed and either embedded in optimal cutting temperature compound (OCT, R.A. Lamb, Eastbourne, U.K.) and frozen in liquid nitrogen, or immersed in 4% PFA overnight and embedded in paraffin. Tissue sections were cut at 30 μm or 5 μm for OCT or paraffin embedded sections respectively and mounted onto slides. Human sections were purchased from US Biomax, Inc.

Hematoxylin and eosin staining was performed according to standard procedures. In brief, paraffin embedded sections were dehydrated and brought to distilled water. All sections were then processed in parallel. Nuclei were stained with Haematoxylin, then rinsed in tap water, then stained with Eosin (2 min), rinsed with tap water, dehydrated and mounted using entallan.

For periodic acid Schiff staining, sections were deparaffinised in xylene and rehydrated using ethanol and distilled water. Samples were immersed in 1% alcian blue and 3% acetic acid (pH 2.5) for 5 minutes then washed and treated with 1% periodic acid for 5 minutes. Samples were then washed again before addition of Schiff’s reagent for 15 minutes. After another wash in distilled water, a wash in tap water and a rinse in distilled water, samples were stained using hematoxylin (HaemQS, Sigma Aldrich) for 1 minute.

#### Trichrome staining

Sections were deparaffinised in xylene and rehydrated using ethanol and distilled water. Samples were re-fixed in Bouin’s solution for 1 hour at 56 °C and rinsed with tap water. Samples were then stained in Weigert’s iron haematoxylin for 10 minutes, rinsed in tap water for a further 10 minutes and washed in distilled water. Following this, samples were stained in Biebrich scarlet-acid fuishin for 10 minutes, washed in distilled water then differentiated in phosphomolybdic-phosphotungstic acid solution for 10 minutes. Sections were then immersed in aniline blue for 5 minutes, rinsed in distilled water and differentiated in 1% acetic acid for 5 minutes. Sections were mounted in entellan.

Immunohistochemical staining was performed using an antibody to GR (1:400, clone M-20, sc-1004, Santa Cruz) or caveolin-1 (1:300, clone N-20, sc-894). Antigen retrieval was performed with Citrate Buffer pH6.0) before primary antibody incubation overnight. For DAB staining, sections were incubated with a biotinylated secondary followed by HRP linked to strepavadin (all Vector Laboratories). Samples were incubated with 3,3′-diaminobenzidine (DAB, Vector Laboratories) dissolved in water, then incubated for 5 minutes with DAB dissolved in water with 3% hydrogen peroxide. After DAB staining, sections were counterstained with Toluidine blue. For immunofluorescence, sections were stained with a secondary

#### Histological scoring

Pathological score of ovalbumin treated mice was performed using a semi-quantitative scoring of lung infiltrates on H&E stained sections, performed by two blinded observers. The mean of the two scores was used in the analysis. Large amounts of infiltrate indicated widespread inflammatory cell infiltrate (3 or more cells deep) around blood vessels and bronchioles. The scoring system ranged from 0 (where no infiltrate was detected), to 5, where over 75% of airways and vessels have large infiltrating regions (3 or more cells deep), and cells found in alveolar space.

PAS staining was quantified by counting individual nuclei in airways and determining the number of PAS stained cells. This was then expressed as a percentage of total number of cells seen.

Following the histological preparation, samples were incubated in permeabilization buffer (TBS, 0.1% v/v Triton-X 100) overnight at 4 °C. Sections were then incubated overnight at 4 °C with primary antibody (Cav1 1:200, clone N20, Santa Cruz) diluted in PBS (pH 7.4, 0.1% v/v Triton-X 100) with goat or horse serum. Sections were washed 3 times in PBS and incubated with fluorophore conjugated secondary diluted in PBS (pH 7.4, 0.1% v/v Triton-X 100) for 2 hours at 4 °C, followed by another 3 PBS washes and incubated over night at 4 °C in another primary (CCSP 1:500, 07–623, Merck Millipore or VE-cadherin sc3458, Santa Cruz) diluted in PBS (pH 7.4, 0.1% v/v Triton-X 100) with goat or horse serum. After washing 3 times in PBS, samples were incubated with an alternate fluorophore secondary antibody diluted in PBS (pH 7.4, 0.1% v/v Triton-X 100) for 2 hours at 4 °C. After 3 washes in PBS, samples were mounted using Vectamount AQ (Vector Laboratories, Peterborough, UK) containing DAPI.

### Brightfield and fluorescence microscopy

Images were acquired on an Axio Imager.A1 (Zeiss) microscope using either a 10x Zeiss EC Plan-NEOfluar or 20x Zeiss EC Plan-NEOfluar objective. Images were collected using AxioCam MRc (Zeiss). Raw images were visualized using AxiovisionRel. 4.7 (Zeiss) and processed using Image J.

### Statistical analysis

Data were analyzed with the graphpad prism software. Results are expressed as mean and standard error, unless otherwise specified. Data were analyzed by 1-way analysis of variance (ANOVA) with the Bonferroni post-hoc analysis, or two-way Student’s t-test. The statistical analysis was conducted at 95% confidence level, with a p value less than 0.05 being considered statistically significant.

## Results

### Caveolin-1 and the glucocorticoid receptor directly interact in the lung *in vivo*

**Figure 1:**
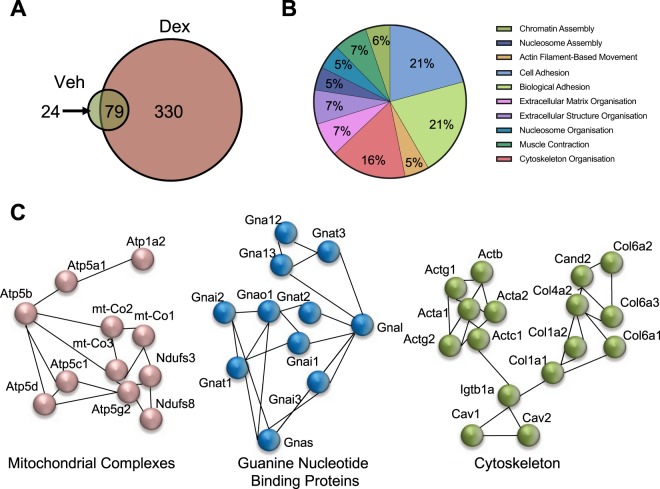
Ligand regulated Glucocorticoid Receptor Lung Interactome. C57BL/6J mice were treated with DEX (1 mg/kg, i.p.) for 1 hour. Lungs were harvested, protein isolated and GR was immunoprecipitated. Resulting immunoprecipitate was analyzed by mass spec for GR interacting proteins (**A**). GO biological process for DEX-induced GR interacting proteins (**B**). GR interacting proteins were analyzed by STRING, and 3 nodes of interest are shown (**C**).

To identify GR-interacting proteins potentially capable of modifying glucocorticoid action in the lung, we isolated lungs from C57BL/6J mice treated with vehicle (cyclodextrin), or a synthetic Gc, dexamethasone (DEX) for 1 hour. Protein was extracted, GR was immunoprecipitated and complexes were subjected to MALDI-TOF mass spectrometry. 103 proteins were identified in vehicle treated lungs, and 409 in DEX treated lungs (Fig. 1A). GR interacting proteins in DEX treated lungs were analyzed by gene ontology, with a large proportion of GR interacting proteins being involved in adhesion, cytoskeletal organization and nucleosome assembly (Fig. 1B). Additionally, GR binding partners were analyzed using STRING^25^, which revealed 7 coherent nodes (Fig. S3), for example mitochondrial complexes, guanine nucleotide exchange proteins and cytoskeletal proteins (Fig. 1C). We have previously identified glucocorticoid regulation of mitochondrial function^24^. However, the cytoskeletal node was of interest as both caveolin-1 and -2 were present, and we had previously found that caveolin-1 is required for the non-genomic glucocorticoid activation of AKT and GSK3β, their downstream effects on cell cycle progression, and that caveoin-1 directly interacts with GR in the A549 human lung epithelial cell line^4^.

### Caveolin-1 knockout alters lung phenotype and increases Gc dependent transactivation

**Figure 2:**
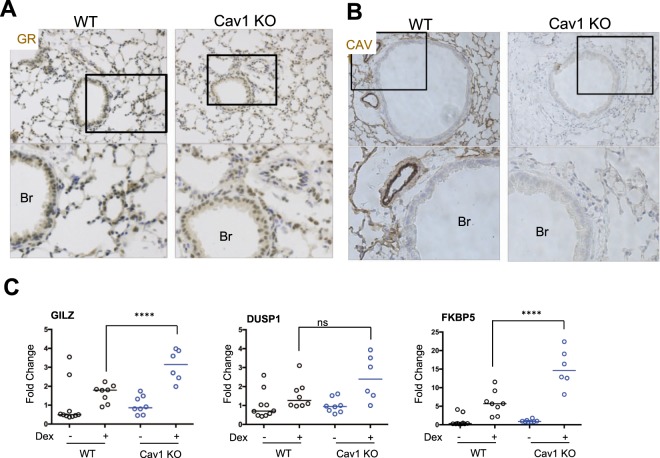
Caveolin-1 Regulates Glucocorticoid Transactivation in the Lung. (**A**) Lungs from WT and Cav1 KO mice were fixed and embedded in paraffin. Lung sections embedded in paraffin were stained for (**A**) GR or (**B**) Cav-1. (**C**) WT and Cav1 KO mice were treated with DEX (1 mg/kg) or vehicle (cyclodextrin) for 6 hours. Lungs were extracted, RNA was harvested and analyzed by qPCR (N = 5–10). Statistical analysis by 2-way ANOVA. p < 0.05*, p < 0.0001**** Images shown at 10x magnification, with highlighted areas at 20x magnification (**A**,**B**). Br is bronchiole. Individual images show different regions of interest.

Lungs from WT and *Caveolin-1* knockout (*Cav1* KO) mice were fixed and immunostained for GR protein and Caveolin-1α (Fig. 2A, B). Cav1α was widely expressed through lung parenchyma, with very high expression localized to vascular endothelium, and a striking absence from the airway epithelial cells (Fig. 2B). This was further supported by immunostaining sections from human lung (Fig. S1B) and dual immunofluorescence of CCSP (club cell secretory protein) a bronchial epithelial cell marker with Cav1α (Fig. S4A), and VECAD (vascular endothelial cadherin) an endothelial marker with Cav1α (Fig. S4B). We found no difference in either levels of GR expression or apparent subcellular localization between genotypes.

To determine the effect of Cav1 on GR transactivation *in vivo*, WT and *Cav1* KO mice were treated with DEX (intraperitoneal, 1 mg/kg) for 6 hours before lungs were recovered, RNA extracted and Gc transactivated genes measured by qPCR. *Gilz* and *Fkbp5* showed an increased response to DEX administration in the *Cav1* KO mice compared to WT, with an increase in *Dusp1* response not reaching statistical significance (Fig. 2C).

Caveolin1 expression is associated with fibrotic lung pathology both in mouse and human. *Cav1* KO mice show lung hypercellularity and fibrosis, and human fibrotic lung samples have reduced caveolin1 expression^27^. Therefore, we turned to *Cav1* null mice and analyzed lung tissue by H&E (Fig. S1E) and Masson’s Trichrome for fibrosis (Fig. S1D). Lung parenchymal tissue showed increased cellularity, and around the airways there was a striking increase in connective tissue deposition.

As GR transactivation was greater in *Cav1* null lungs, we postulated that *Cav1* null mice would be more responsive to Gc suppression of inflammation, as transactivation is a key component of the anti-inflammatory actions of Gcs^14,28,29^.

### Caveolin-1 facilitates the innate immune response in the lung through TLR4, but does not affect the Gc suppression of inflammation

**Figure 3:**
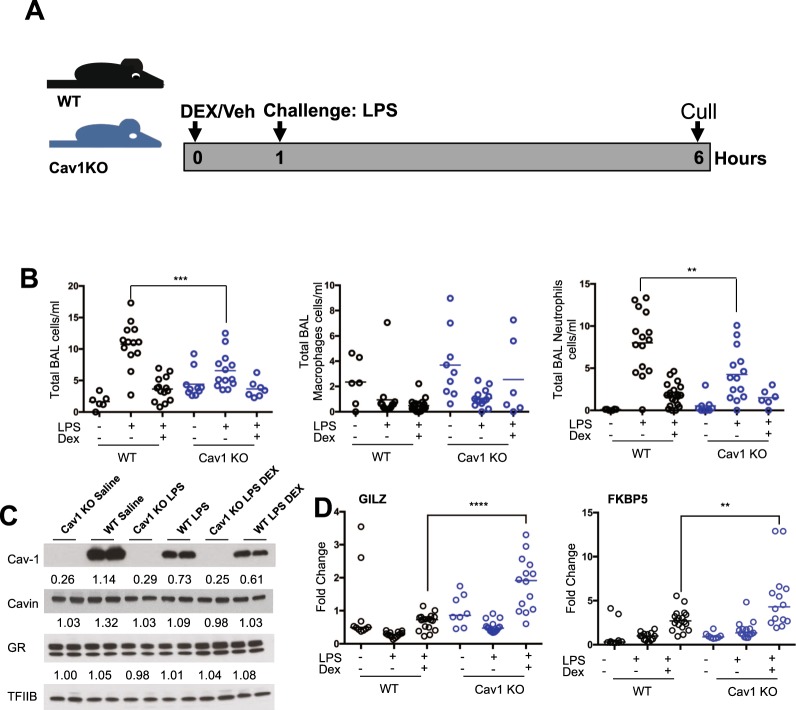
Caveolin-1 Regulates LPS Response in the Lung. (**A**) WT and Cav1 KO mice were treated with saline or dexamethasone (1 mg/kg) for 1 hour before being exposed to aerosolized saline or LPS (1 mg/ml) for 20 minutes before being left for 5 hours. Animals were then sacrificed, and lung cell infiltrate was removed by bronchoalveolar lavage. (**B**) Cells were counted and stained for cell specific markers or counted by cytospin, and macrophages and neutrophils were quantified (N = 6–20). (**C**) Protein was extracted from whole lungs and analyzed by western blot, representative image shown. Values indicate densitometry compared to TFIIB loading control, averaged between 2 replicates. (**D**) RNA was isolated from whole lung, and transactivated genes were analysed by qPCR (N = 8–18). Data shown as median with individual data points. Statistical analysis via 2-way ANOVA. p < 0.05*, p < 0.01**, p < 0.001***.

To examine the impact of Cav1 on the anti-inflammatory actions of Gc in the lung mice were challenged with nebulized lipopolysaccharide (LPS), a TLR4 ligand (Fig. 3A). Both WT and *Cav1* KO mice display an increase in total immune cell infiltration to the bronchoalveolar space after LPS treatment, and a repression with DEX. However, fewer neutrophils were recovered from LPS-treated *Cav1* null mouse lungs compared to WT animals (Fig. 3B). There was however no difference in the Gc dependent inhibition of immune cell infiltration into the lung upon LPS treatment between the genotypes. This suggests that despite the increase in the transactivation potential of GR in the absence of Cav1, the ability of Gcs to repress immune cell infiltration into the lung, and Gc regulation of cytokine production remains unchanged, indicating that GR mediated transpression of NFκB and AP-1 is key to the action of Gcs in this model.

**Figure 4:**
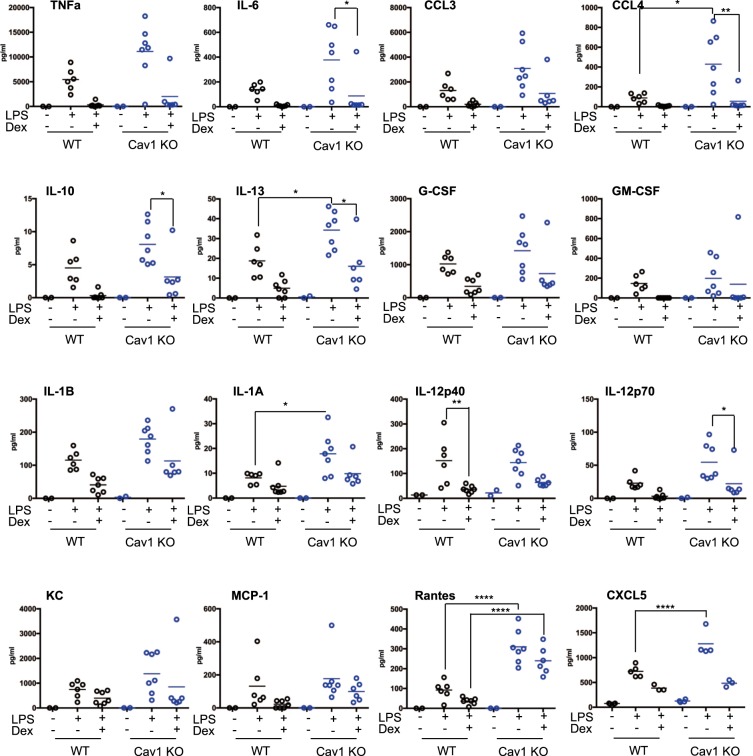
Caveolin-1 Regulates BAL Cytokine Production. (**A**) WT and Cav1 KO mice were treated with saline or dexamethasone (1 mg/kg) for 1 hour before being exposed to aerosolized saline or LPS (1 mg/ml) for 20 mins and left for 5 hours, BAL was harvested and analyzed by multiplex for cytokine production. Data shown as median and individual data points (N = 2–7). Statistical analysis via Mann-Whitney test. p < 0.05*, p < 0.01**, p < 0.001***, p < 0.0001****.

Lung GR expression levels were similar between genotypes, and unaffected by LPS or LPS + DEX, when compared to the loading control. Cav1α expression was completely absent from *Cav1* KO lung. Cavin, another major part of caveolin based lipid rafts, showed no change in expression, however LPS exposure significantly decreased Cav1α expression (Figs 3C or S6). To determine whether LPS altered the increased transactivation potential of Gcs in *Cav1* KO mice, we assessed *Gilz* and *Fkbp5*, the two highly responsive genes when treated with DEX only (Fig. 3D). Even though *Cav1* KO mice showed increased Gc transactivation of *Gilz* and *Fkbp5* this did not affect Gc repression of inflammation. To explore the impact on cytokine and chemokine secretion, BAL fluid was analyzed for a broad panel of cytokines. Despite seeing no effect of *Cav1* on Gc mediated suppression of cytokine secretion, we did find an interesting discrepancy between gene expression and cytokine production after LPS in WT and *Cav1* null lungs. IL-6, CCL4, IL-10, IL-13, IL-1α, IL-12p40 and p70, RANTES and CXCL5 all showed differential responses in BAL isolated from *Cav1* KO mice after LPS challenge (Fig. 4). Again, these inflammatory mediators were similarly repressed by GR in both genotypes. The somewhat exaggerated cytokine production in the *Cav1 KO* mice did not affect neutrophilic infiltrate as expected, and this may be due to migratory defects in caveolin-1 deficient neutrophils, as previously described^30^ which provides an explanation for the discordant results. Further analysis of isolated alveolar macrophages treated with LPS showed an increased response to LPS in *Cav1* KO alveolar macrophages (Fig. S5). However, as with the mice, GC exposure reduced expression of *Ccl3* and *Ccl4* in both genotypes equally.

### Caveolin-1 does not modify Gc suppression of allergic inflammation in the lung

**Figure 5:**
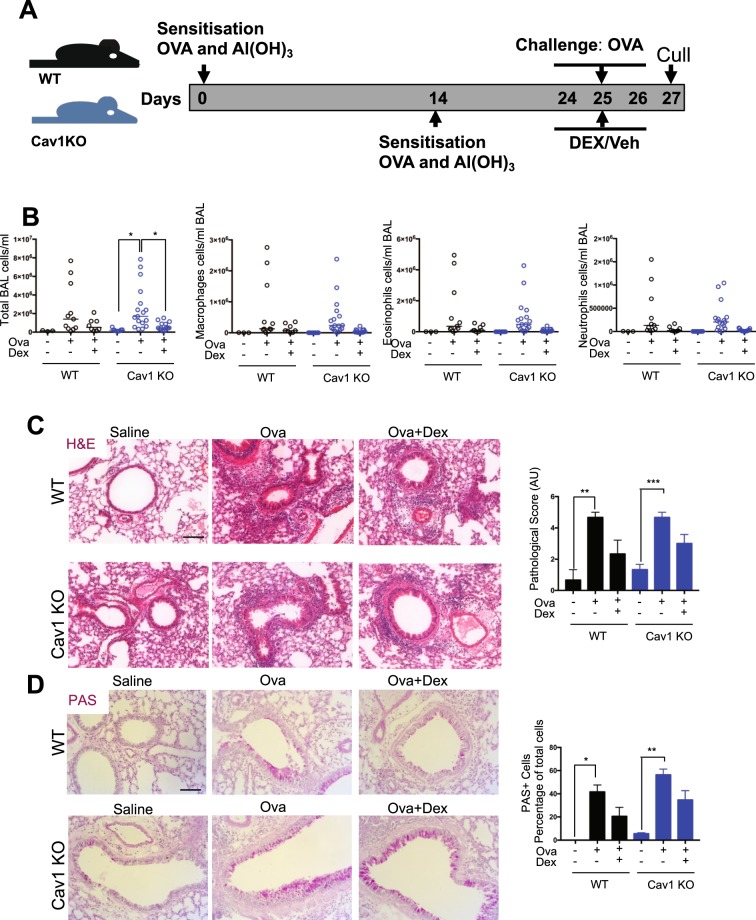
Caveolin-1 is does not affect glucocorticoid repression in a murine model of asthma. (**A**) WT and Cav1 mice were sensitised to ovalbumin (OVA) with Al(OH)_3_ adjuvant on days 0 and 14. Mice were then treated with DEX (1 mg/kg) or vehicle (cyclodextrin) I.P. 3 hours before challenge with OVA (I.N.) on days 24, 25 and 26. Mice were then culled on day 27. (**B**) BAL was harvested and lungs were fixed in 4% PFA before embedding in paraffin. BAL was analyzed for total cell number, macrophages, neutrophils and eosinophils (N = 3–17). (**C**) Fixed lungs were stained by H&E, or (**D**) PAS and quantified (N = 3–7). Data shown as median with individual data points (**B**), or mean + SEM (**C,D**). Statistical analysis by 2-way ANOVA. p < 0.05*, p < 0.01**, p < 0.001*** Scale bar 100 µm.

As acute challenge had revealed an impact of caveolin on elaboration of a neutrophilic inflammatory response we then moved to study an allergic pulmonary response which involves a different set of immune cells including eosinophils and mast cells. WT and *Cav1* KO mice were sensitized with i.p. ovalbumin (OVA) and aluminium hydroxide adjuvant on days 0 and 14, before three intranasal (i.n.) challenges of OVA on consecutive days (Fig. 5A). BAL fluid was analyzed for total cellular infiltrate after cull.

Ovalbumin challenge significantly increased the total immune cell count in BAL - macrophages, eosinophils and neutrophils in the *Cav1* null mice, but with a wide variation in response (Fig. 5B). Lungs were isolated, fixed, and stained by H&E, showing an increase in inflammation, and a similar repression by Gc in both genotypes (Fig. 5C). Lungs were also stained using Periodic acid Schiff staining to identify goblet cells (Fig. 5D) Histopathological scoring did not find any significant difference between WT or *Cav1* null responses, in either the inflammatory response to ovalbumin, or the anti-inflammatory actions of Gcs (Fig. 5C,D).

### Immunosuppression of *T. muris* infection by Gc is not dependent on Caveolin-1

**Figure 6:**
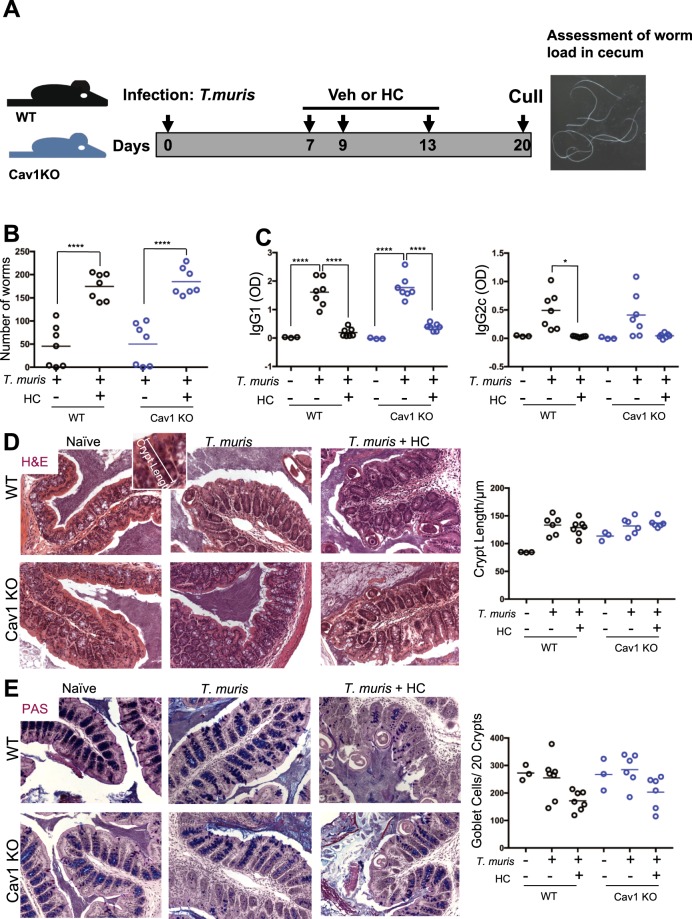
Caveolin-1 is Dispensable during Trichuris Muris Infection. (**A**) WT and Cav1 KO mice were treated with 200 *T. muris* eggs (1 mg/kg oral gavage). On days 7, 9 and 13, mice were treated with either vehicle (saline) or hydrocortisone (5 mg/kg). (**B**) Animals were sacrificed on day 20, cecae were removed, and worm burden assessed (N = 7). (**C**) Thymuses were isolated and treated with *T. muris* antigen over-night. Supernatant was removed and analyzed for IgG1 and IgG2c by ELISA (N = 3–7). (**D**) Large intestine were isolated and sections were stained for H&E and crypt length determined or (**E**) PAS and goblet cells per 20 crypts counted (N = 3–7). Images taken at 10x magnification. Data shown as median with individual data points. Statistical analysis by 2-way ANOVA p < 0.05*, p < 0.01**, p < 0.001***, p < 0.0001****.

The lung and gastrointestinal tract epithelia share common features; they both directly interface with the environment, and contain a variety of different cell types; such as epithelia, mucin producing cells, smooth muscle, resident macrophages and other immune cells. Gc treatment is also frequently used to repress inflammatory bowel diseases, such as Crohn’s disease^31^. Intestinal Th2 adaptive immune responses are required for effective responses to parasitic infestations of the gut, such as with the gut-dwelling nematode parasite *Trichuris muris*. Gc treatment impairs these Th2 responses and permits the parasitic worms to evade expulsion from the gut. The allergic response tested in the lung, is also Th2 dominant. Therefore, we tested anti-*T. muris* responses in the *Cav1* null mice. We used *Trichuris muris* (*T.muris*), which infects the cecum of mice. Mice were infected with *T.muris* eggs (200 eggs by oral gavage) and dosed with Gc (hydrocortisone 50 mg/kg, i.p.) or saline on days 7, 9 and 13, then culled on day 20 post infection (Fig. 6A). The large intestine was analyzed by IHC for GR expression and localization (Fig. S1C). GR is mostly localized in the mucosal epithelial cells, with less in the interstitial space and smooth muscle. The localization of GR was not different between the genotypes. As with the lung, caveolin-1α expression was devoid from the epithelium, and was mostly localised to the smooth muscle, and cells in the interstitial space (Fig. S1D).

Mice infected with worms and treated with saline showed a significant reduction in worms within 20 days, consistent with the development of a Th2 immune response. Gc treatment impaired the immune response resulting in retention of worms (Fig. 6B). This was unaffected by genotype. Quantification of antibody titre from cultured thymocytes isolated post-mortem from WT or *Cav1* KO mice demonstrated a significant increase in worm antigen-specific IgG1, but a non-significant increase in IgG2c upon infection. IgG1 was significantly suppressed by hydrocortisone in both WT and Cav1 KO mice, without genotype differences, but due to the small increase in IgG2c, we did not detect a significant reduction in the Cav1 KO mice (Fig. 6C).

Assessment of damage induced within the gut by immune cells during the course of infection was performed using immunohistochemistry. Crypt length (Fig. 6D) and goblet cell number (Fig. 6E) show no difference between the genotypes upon infection, or after hydrocortisone treatment.

## Discussion

The ubiquitously expressed glucocorticoid receptor plays an essential role in both energy metabolism and regulation of immunity. Its target genes vary by cell type, with a clearer understanding emerging of how cell-type determining factors prime target sites in the genome, to confer cell-type specificity of action. In addition, there is evidence that cell state or environment can also determine GR target gene engagement, allowing glucocorticoid signaling to be highly specific to context^8^. However, in addition to chromatin, GR interacts with many other proteins in the nucleus and cytoplasm, and these interactions offer the opportunity to regulate GR function, or for GR to regulate their function^4,32^. In this work we use an unbiased proteomic approach to find GR partner proteins in a key therapeutic Gc target tissue, the lung. We identify the membrane lipid raft protein caveolin-1 as an interacting partner, and define a distinct, and restricted role for caveolin-1 in regulating GR transactivation under homeostatic conditions. We extend these studies into *in vivo* Gc responses and find a surprisingly specific impact of caveolin-1 on acute inflammation, but not its regulation by Gc. In further studies we find no impact of caveolin-1 expression in allergic lung inflammation, or on the effect of Gc repression of inflammation. To test the role of caveolin-1 in another model of epithelial Th2 immunity we tested *T.muris* infestation; and again saw no caveolin-1 phenotype either basally, or in response to Gc challenge.

The application of unbiased protein interactome screening offers new insights into GR function. As tissue types differ in their response to Gc we used mouse lung as our tissue of choice, as it offers a number of important therapeutic indications, as well as well-defined *in vivo* models. The interactome study revealed three major ontological terms, which included cytoskeleton. This was of particular interest as caveolin-1 had previously been found as a GR modulating protein in a lung cancer cell line^4^, and caveolin-1 is essential for normal lung function^22^.

*In vivo* studies revealed increased GR transactivation in the caveolin-1 null lung. Two of the transactivated genes, *Gilz* and *Dusp1*, have been shown to mediate the anti-inflammatory actions of Gcs^29,33–35^, and so our findings suggested that the loss of caveolin-1 might increase the anti-inflammatory efficacy of Gc. This would be of interest, as partial, or negligible responses to Gc underlie most human lung inflammatory disorders, such as COPD^36,37^. Therefore, we tested the impact of acute LPS challenge on the caveolin-1 null mice.

Nebulised LPS offers an acute localized pulmonary inflammatory challenge. We observed reduced inflammatory neutrophil responses in the caveolin1 null animals, which may reflect the previously noted requirement for caveolin-1 in neutrophils to permit efficient transendothelial migration^30^, but no effect of *Cav1* on Gc suppression of immune cell infiltration. While we similarly observed no effect of *Cav1* on the regulation of cytokines by Gcs, we did note that in the *Cav1* null mice there was reduced expression of a number of cytokines and neutrophil chemokines genes in whole lung, suggesting a more widespread effect, and that the impaired neutrophil migration seen in the Cav1 null mice may also result from reduced chemokine gradients. However, the major neutrophil chemokine *Cxcl5* was found to be significantly increased in the caveolin-1 null lungs, which was surprising as Cav1 had been implicated in NF-κB responses to inflammation^19^, and *Cxcl5* is a well-characterised NF-κB target gene. Taken together, loss of Cav1 results in impaired elaboration of inflammatory responses in the lung, and loss of inflammatory signal amplification. This, coupled with the neutrophil chemotactic defect previously identified in response to loss of Cav1^30^, results in the blunted neutrophilic inflammatory response to nebulised LPS challenge. However, we did not see any evidence that the action of Cav1 affected glucocorticoid regulation of the pulmonary inflammatory response.

The inflammatory phenotype observed in response to innate immune challenge with LPS is mediated by a neutrophilic response to an inflammatory reaction initiated mainly by macrophages^26^. Therefore we sought to determine caveolin-1 dependent Gc responses in an alternate model of lung inflammation, involving other major immune cell types. To this end, we used the ovalbumin sensitisation and challenge model, which involves a Th2 skewed adaptive immune response, with contributions from recruited eosinophils. This mouse model reflects some aspects of the allergic inflammatory reaction seen in human asthma, and so is relevant because as Gcs are a common, and effective treatment for asthmatic conditions, and are also highly effective in the OVA sensitisation and challenge model. There was no genotype effect on the ovalbumin response, or on its inhibition by dexamethasone. Histological examination did not detect any differences in immune cell infiltration, or goblet cell hyperplasia. To test Th2 responses further, in a distinct epithelial setting we used intestinal infection with the *T.muris* helminth. This infection provokes a Th2-polarised response, which is essential for worm expulsion, and the resulting immune response is Gc sensitive. Rather like the ovalbumin challenge to the lung we did not see a genotype effect. In both the lung, and gut, we saw very little caveolin1 expression in the epithelium, but caveolin1 is expressed in the myeloid lineage, a key part of the initial pathogen response in both tissues. There are two splice variants of the Cav1 gene, Cav1α, and Cav1β. In our studies we did not distinguish between them, as the mass spectroscopy data did not resolve the difference, and the Cav1 null mouse loses expression of both variants. It may be of interest in the future to determine if either of the variants plays a dominant role in the GR interaction. Our findings demonstrate that Gc anti-inflammatory actions on immune cell infiltration, cytokine production, worm load and T cell response, are independent of Cav1. Cav1 and the GR interact, and Cav1 affects the induction by the activated GR of a number of important anti-inflammatory genes. However, Cav1 did not play a role in modulating Gc regulation of either acute, or chronic inflammatory responses in the lung, or the gut.

## Supplementary information

Supplementary figures

Identified proteins via mass spec and GO analysis
