# Supplementary material for: Caveolin1 interacts with the glucocorticoid receptor in the lung but is dispensable for its anti-inflammatory actions in lung inflammation and Trichuris Muris infection
Source: Sci Rep. 2019 Jun 12;9:8581. doi: 10.1038/s41598-019-44963-0 (PMC6562044; doi:10.1038/s41598-019-44963-0)
Supplement: Supplementary file 1 — Supplementary figures [file 41598_2019_44963_MOESM1_ESM.pdf]

Caveolin1 interacts with the glucocorticoid receptor in the lung but is dispensable for its anti-inflammatory actions in lung inflammation and Trichuris Muris infection

Caratti G[1,**], Poolman T[1], Hurst RJ[1], Ince L[1,##], Knight A[1], Krakowiak K[1], Durrington HJ[1], Gibbs J[1], Else KJ[1], Matthews LC[3,*], Ray DW[1,2,4,*]

[1]Faculty of Biology, Medicine, and Health, University of Manchester and Manchester Academic Health Sciences Centre, Manchester M13 9PT, UK.

[2]Department of Endocrinology, Manchester Royal Infirmary, Manchester University Foundation Trust, Manchester, M139WL, UK

[3]Leeds Institute of Cancer and Pathology, Faculty of Medicine and Health, University of Leeds, Leeds, LS9 7TF, UK.

[4]OCDEM, University of Oxford, Oxford, UK

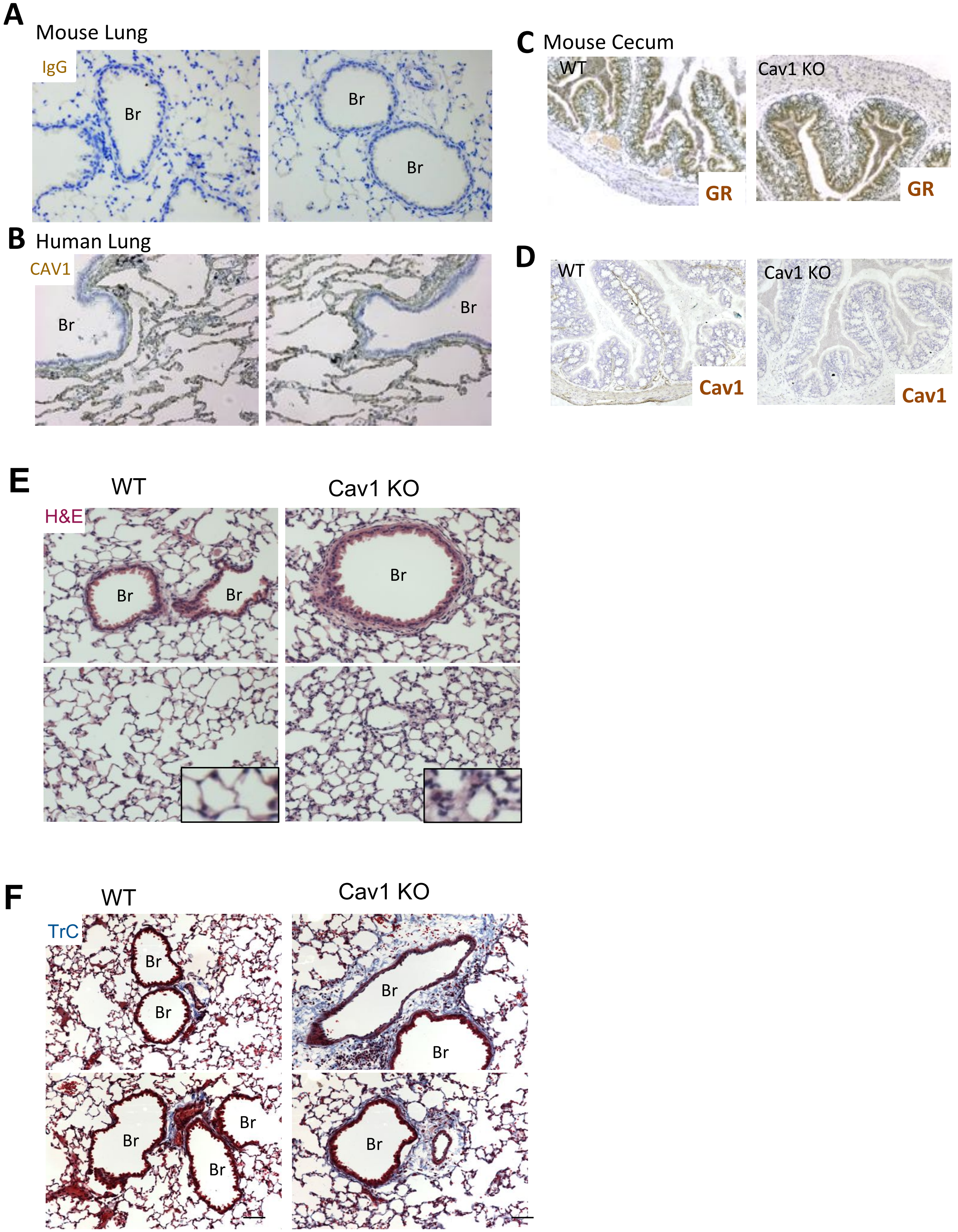

Supplementary Figure 1. Lungs from WT mice and Cav1 KO mice were fixed and embedded in paraffin. Sections were stained using an IgG control (**A**). Human lung sections were also stained for caveolin-1 in brown (**B**). Mouse large intestine was stained for GR (**C**) or Cav1 (**D**) in brown. (**E**) Lung sections from WT and Cav1 KO mice were stained for H&E and (**F**) Masson's Trichrome. Views show representative area, or areas of interest. Original magnification at 10x. Insets at 3x zoom of original.

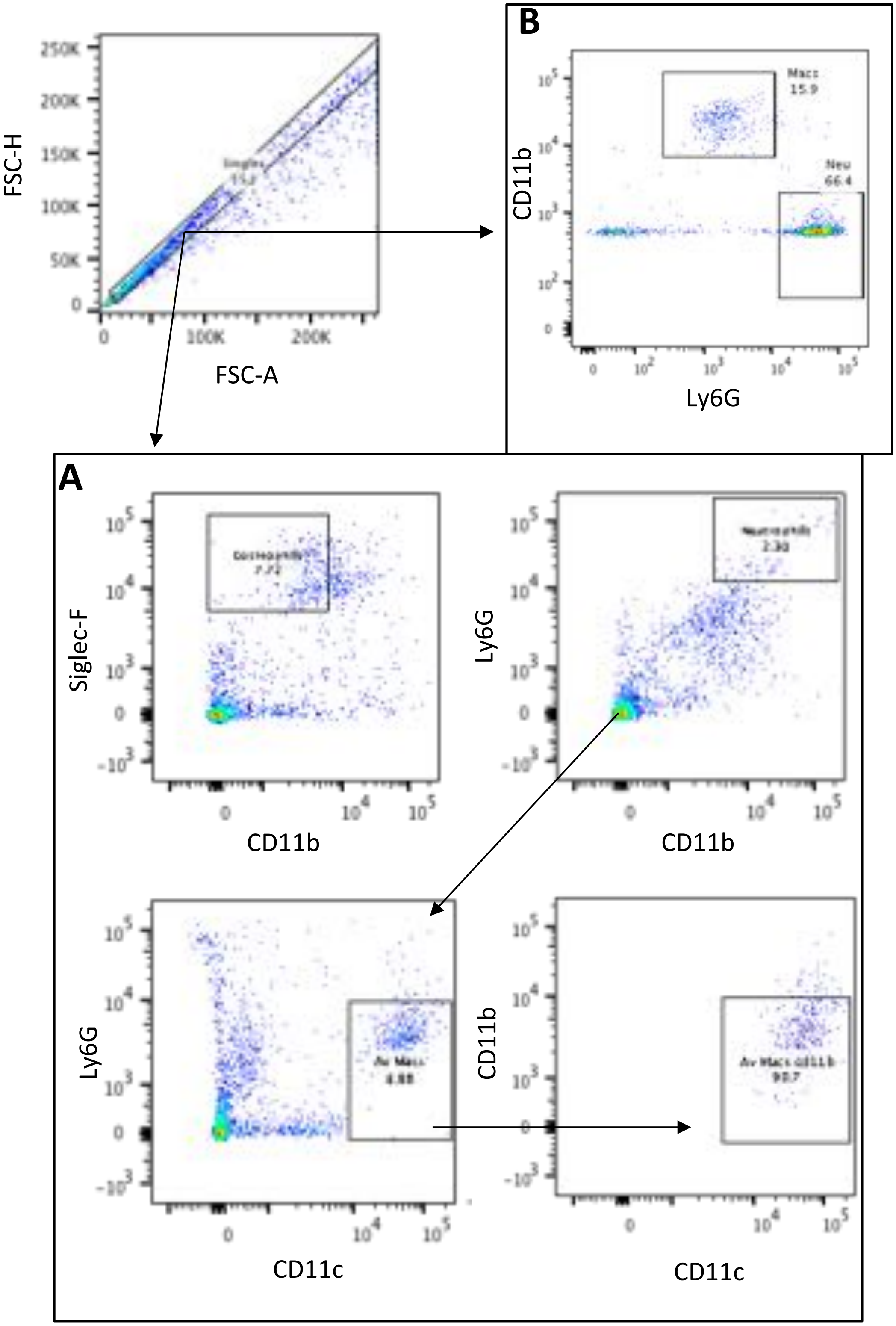

Supplementary Figure 2. BAL from WT and Cav1KO mice was FC blocked, then FACS sorted. Gating strategy for ovalbumin challenged BAL (**A**). Gating strategy for LPS challenged BAL (**B**).

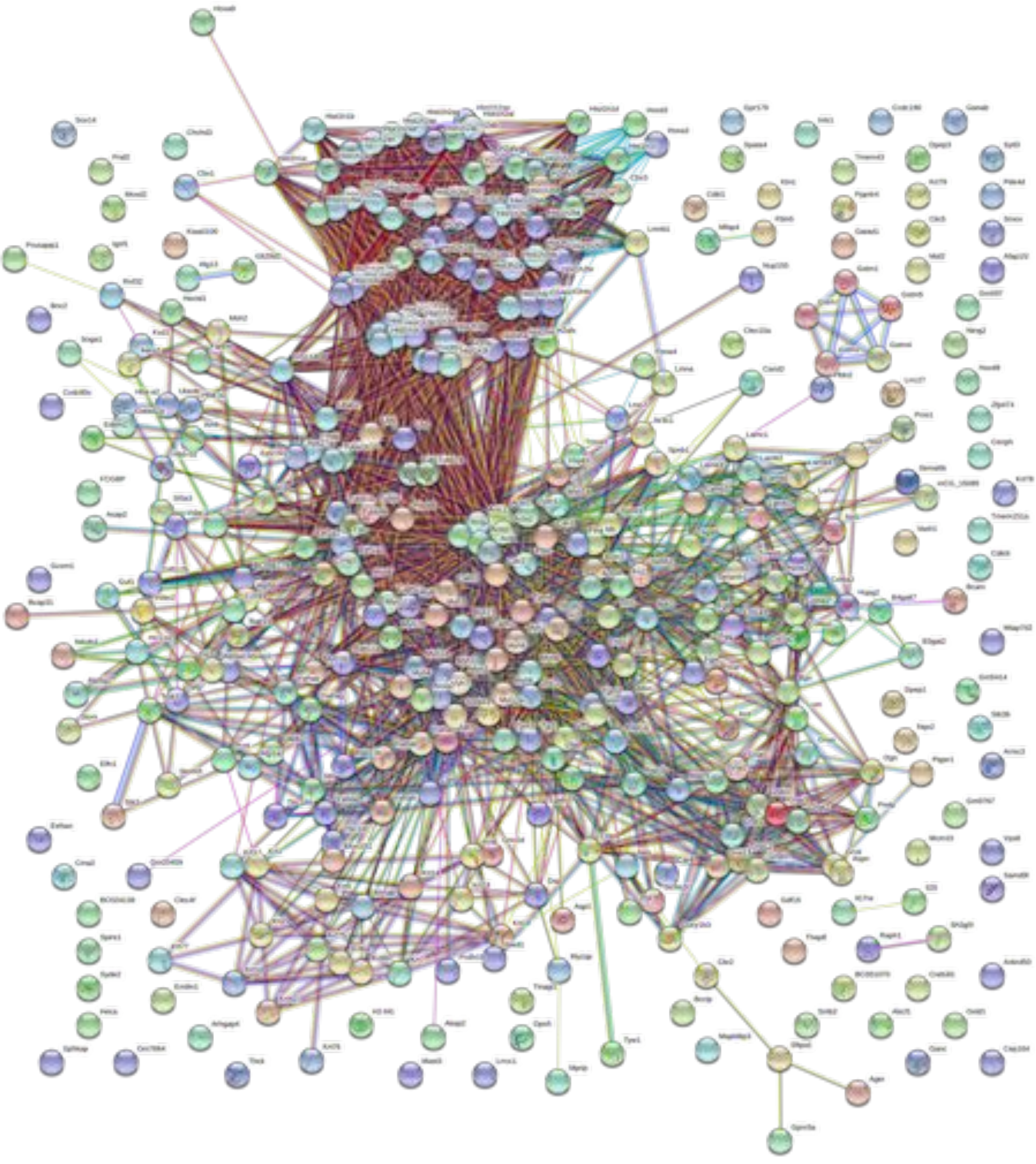

Supplementary Figure 3. Raw output from String.

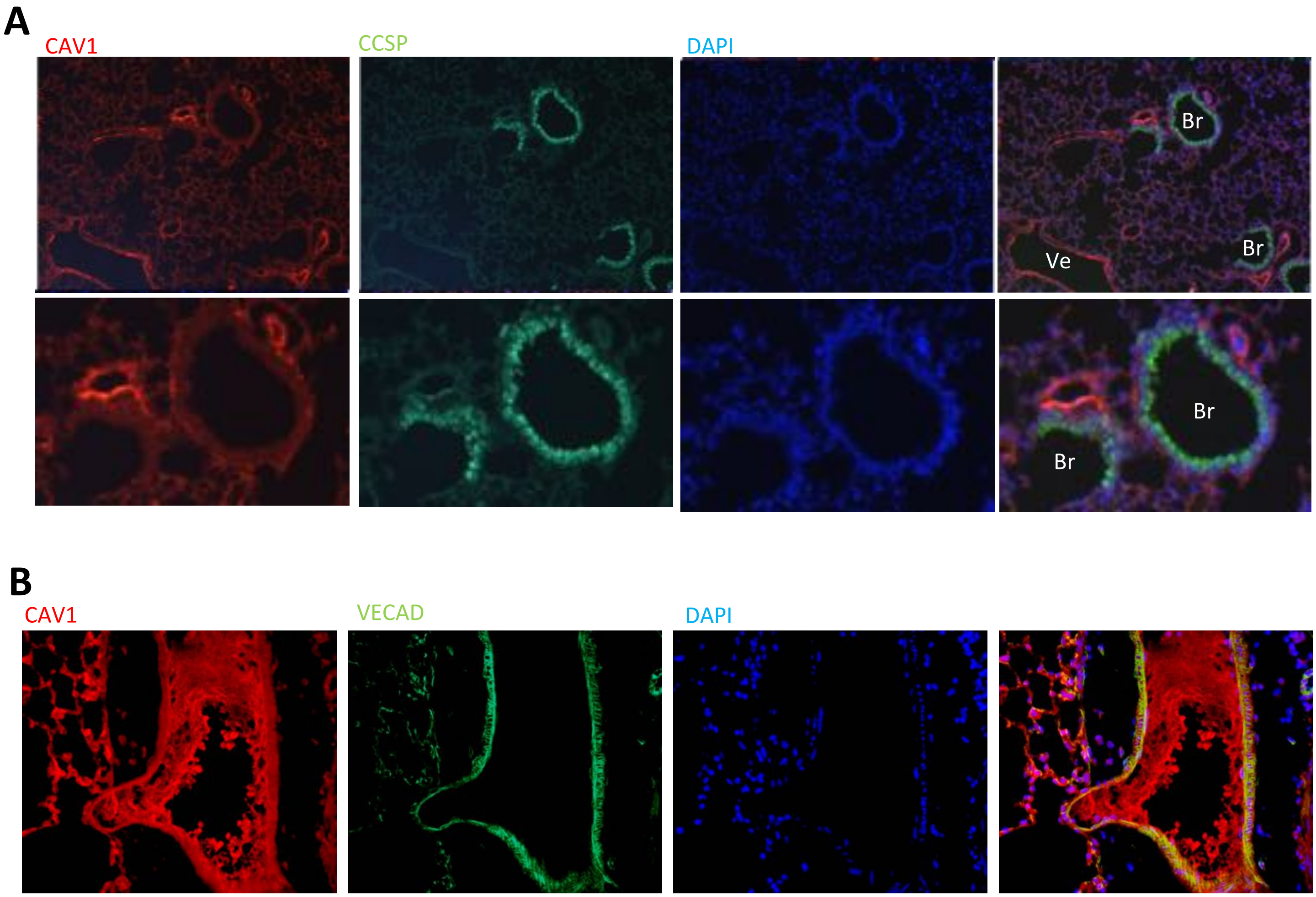

Supplementary Figure 4. Caveolin-1 is not Expressed in Bronchial Epithelial Cells
(**A**) Lungs from C57BL/6J mice were fixed and embedded in paraffin. Sections were costained for caveolin-1 (red), clara cell secretory protein (green) and nuclei were stained with DAPI (blue). (**B**) Lungs were constained for caveolin-1 (red), VECAD (green) and nuclei stained with DAPI (blue). The merge shows cellular localisation of each protein. Original magnification 10x (A) or 20x (B).

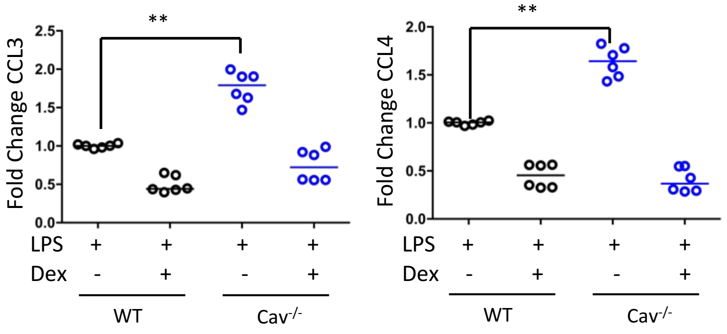

Supplementary Figure 5. Caveolin-1 regulates LPS responses in alveolar macrophages.

Alveolar macrophages were isolated from naïve WT and Cav1 KO mice, cultured over night then washed of non-adherent cells. Final adherent cells were treated with LPS (100ng/ml) and/or dex (100nM) for 6 hours. RNA was extracted and analysed by qPCR. Statistical analysis via 1-way ANOVA. $p < 0.01$**

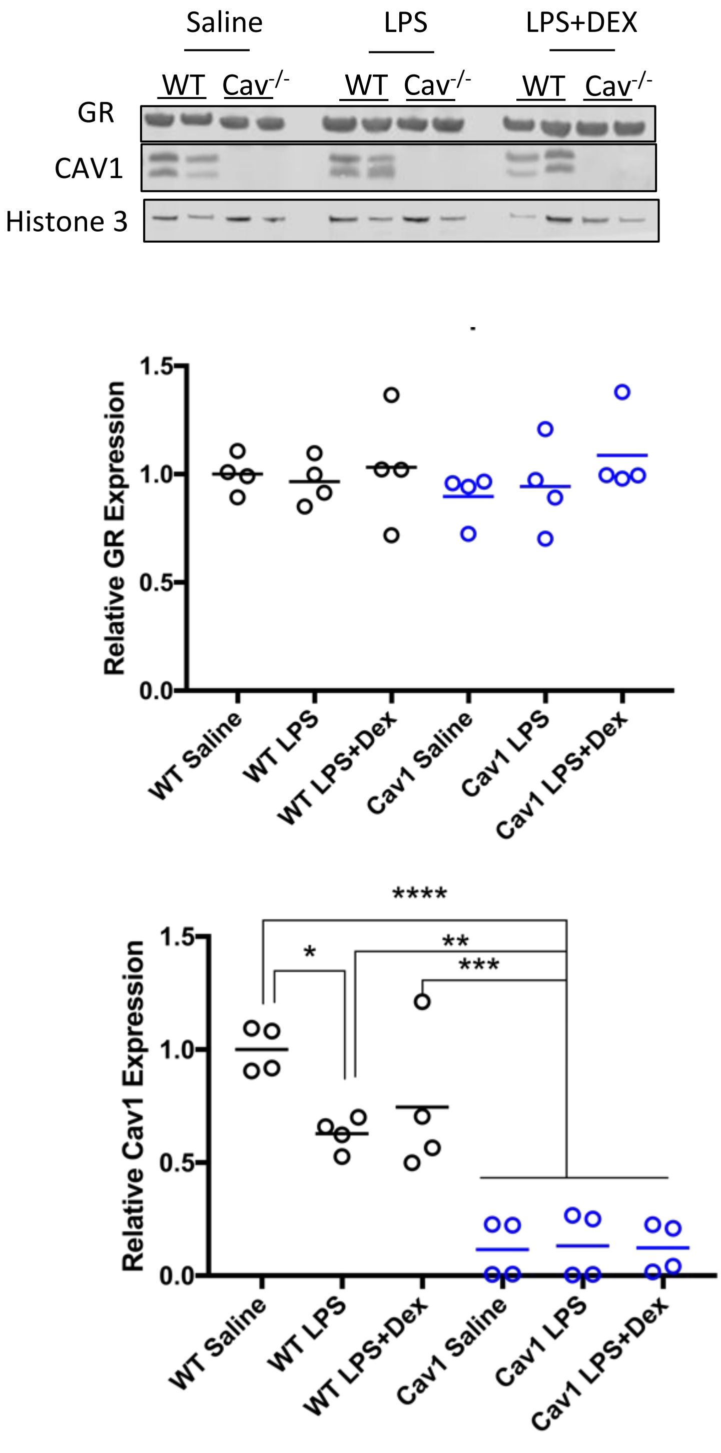

Supplementary Figure 6. Caveolin-1 and GR expression during LPS and dex exposure Protein was extracted from whole lungs and analyzed by western blot. Data from two independent blots were normalized to control (WT veh). Statistical analysis by 1-way ANOVA with a Tukey post-hoc test.
